# Annual Meeting of the International Society of Cancer Metabolism (ISCaM): Metabolic Networks in Cancer

**DOI:** 10.3389/fphar.2017.00411

**Published:** 2017-07-04

**Authors:** Nicola Baldini, Angelo De Milito, Olivier Feron, Robert J. Gillies, Carine Michiels, Angela M. Otto, Silvia Pastoreková, Stine F. Pedersen, Paolo E. Porporato, Pierre Sonveaux, Claudiu T. Supuran, Sofia Avnet

**Affiliations:** ^1^Rizzoli Orthopaedic InstituteBologna, Italy; ^2^Department of Biomedical and Neuromotor Sciences, University of BolognaBologna, Italy; ^3^Department of Oncology-Pathology, Karolinska InstituteStockholm, Sweden; ^4^Pole of Pharmacology, Institut de Recherche Expérimentale et Clinique (IREC), Université catholique de LouvainBrussels, Belgium; ^5^H. Lee Moffitt Cancer Center and Research Institute, TampaFL, United States; ^6^Namur Research Institute for Life Sciences (NARILIS), University of NamurNamur, Belgium; ^7^Munich School of Bioengineering, Technical University of MunichMunich, Germany; ^8^Biomedical Research Center of the Slovak Academy of SciencesBratislava, Slovakia; ^9^Department of Biology, Section for Cell Biology and Physiology, University of CopenhagenCopenhagen, Denmark; ^10^Department of Molecular Biotechnology, Molecular Biotechnology Center, University of TorinoTurin, Italy; ^11^Neurofarba Department, University of FlorenceFirenze, Italy

**Keywords:** tumor metabolism, proton dynamics, tumor microenvironment, cancer imaging, cancer therapy

## Abstract

Cancers are metabolic entities wherein cancer cells adapt their metabolism to their oncogenic agenda and microenvironmental influences. Metabolically different cancer cell subpopulations collaborate to optimize nutrient delivery with respect to immediate bioenergetic and biosynthetic needs. They can also metabolically exploit host cells. These metabolic networks are directly linked with cancer progression, treatment resistance and relapse. Conversely, metabolic alterations in cancer are exploited for anticancer therapy, imaging and personalized medicine. These topics were addressed at the 3rd annual meeting of the International Society of Cancer Metabolism (ISCaM) in Brussels, Belgium, on 26–29 October 2016.

## Introduction

Cancers have a high metabolic plasticity allowing cancer cells to survive and proliferate in extreme microenvironmental conditions often characterized by hypoxia, declining metabolic resources and acidosis. Over recent years, experimental and clinical evidence increasingly suggested that metabolic plasticity contributes to tumor progression. It is central to different networks, not only those directly pertaining to metabolism (including autophagy, cell cannibalism, cooperativity and commensalism), but also others referring to broader aspects of cancer cell biology, including genetics, epigenetics, intra- and inter-cellular signaling and the response of tumors to different anticancer therapies. Consequently, novel therapeutic targets have been identified and new treatments are being developed aimed at selectively targeting well-identified metabolic determinants that control phenotypes associated to tumor progression. These phenotypes notably include cancer stemness, cancer cell migration and invasion, metastasis, angiogenesis, cancer cell resistance to anticancer treatments and immunity.

To address these important aspects of tumor biology, the International Society of Cancer Metabolism (ISCaM^1^) was founded in 2014 as a logical expansion of the International Society for the Study of Proton Dynamics in Cancer (ISPDC) that operated from 2009 to 2014. ISCaM’s scope is to improve communication and to foster collaboration between scientists engaged in acidity, proton dynamics, metabolism and microenvironment in cancer research. On October 26–29th 2016, ISCaM hold its 3rd annual meeting in Brussels, Belgium. With a focus on “Metabolic Networks in Cancer,” this multidisciplinary symposium allowed to exchange most recent knowledge related to cancer metabolism between fundamental researchers and clinicians, scientists of international reputation and young investigators and ISCaM members and the international scientific community. It was endorsed by the European Organization for Research and Treatment of Cancer (EORTC) and by the Belgian Télévie.

During the opening lecture, Douglas Hanahan reported on metabolic evasion to anti-angiogenic therapy. When a specific pro-angiogenic pathway is inhibited, the antitumor response is typically transitory due to compensation by alternative pro-angiogenic signals, protection by perivascular macrophages as well as invasion and metastasis. Together with increased autophagy, the engagement of cancer cells in a metabolic symbiosis based on swapping lactate for glucose by glycolytic cancer cells (Sonveaux et al., 2008) was identified as a fourth mode of resistance (Allen et al., 2016). Metabolic plasticity was at the core of ISCaM2016 and was addressed in 11 focused sessions. Oral sessions were accompanied by solid and informative poster sessions.

## General Metabolism and Metabolomics

Cancers with inactivating mutations of genes encoding metabolic enzymes accumulate metabolites affecting signaling and supporting aggressive phenotypes. Christian Frezza discussed the oncogenic role of fumarate, which accumulates when fumarate hydratase (FH) is inactivated. He presented results concerning FH-deficient renal cancer cells, found to undergo a fumarate-dependent epithelial-mesenchymal transition. The talk by Rodrigue Rossignol was focused on metabolic consequences of oncogenic HRAS activation. Multidisciplinary analyses revealed upregulation/activation of several TCA cycle enzymes. Metabolomics showed a reversal of the Warburg effect and increased consumption of glutamate. Aspartate/glutamate carrier AGC2 was proposed as a target in cells with HRASG12V mutation. Denis Buurman presented the Seahorse technology for measuring cellular bioenergetics, explaining how its embedding in Agilent mass spectrometry created a synergy useful at cellular/molecular levels. Other presentations introduced phosphoglycolate phosphatase as a metabolite repair enzyme required for the coexistence of glycolysis and the pentose phosphate pathway (PPP; by Guido T. Bommer), the role of lactate dehydrogenases (LDHs) in metabolic reprogramming of cancer cells and the necessity to target both isoforms for tumor growth inhibition (by Maša Ždralevič), characterization of metabolism in complex cancer cell populations (by Irena Roci), and metabolic profiling of complex tissues for metabolic phenotyping (by Shonagh Russell).

## Amino Acid Metabolism

EACR-sponsored lecturer, Simone P. Niclou, focused on isocitrate dehydrogenase 1 (IDH1) mutations present in >80% of diffuse lower grade gliomas. IDH1 is involved in glutamate and fatty acid synthesis, and regulates cellular redox balance. Alterations of unrecognized metabolic pathways conferring growth advantages were reported. Angela M. Otto then demonstrated aberrant metabolism regulating the malignant potential of cancers. Her team observed high glutaminolysis combined with high glycolytic flux providing more anabolic metabolites to highly metastatic compared to lowly metastatic breast cancer cells. Mutation of p53 and/or high constitutive c-Myc activity explain these differences. Hypoxia is the main microenvironmental feature exerting metabolic effects in tumors. Milica Vucetic highlighted that maintenance of cellular redox equilibrium is essential for cell proliferation under hypoxia. Genetic disruption of the cystine importer xCT inhibited cell proliferation to a higher extent under hypoxia than in normoxia. xCT was marked as a promising target. Citrin, a malate-aspartate mitochondrial shuttle, and argininosuccinate synthase 1 (ASS1) are other possible targets since they interfere with the NAD^+^/NADH ratio. Shiran Rabinovich showed that ASS1 deficiency in patients with citrin upregulation increases cytosolic aspartate availability, while sustaining DNA synthesis/proliferation. These studies exemplified strategies for designing anticancer therapies based on metabolic aberrations in cancer cells.

## Lipid Metabolism

Almut Schulze introduced the concept that cancer cell survival in hostile conditions with limited nutrients relies on enhanced macromolecule biosynthesis from lipids, which is regulated by oncogenic signaling. Major discoveries in this field support the identification of novel potential therapeutic approaches. She showed that sterol regulatory element binding proteins (SREBPs) are controlled by the Akt/mTORC1 signaling axis. The interlacement of metabolism and signaling regulation was also discussed in endothelium and ovarian cancer. Francisco Morales-Rodriguez indeed showed that multifunctional enzyme fatty-acid synthase (FASN) modulates vessel sprouting via mTOR malonylation. Thomas W. Grunt further reported that FASN inhibition interferes with receptor-PI3K-mTORC1 signaling, with subsequent lipid redistribution toward storage, diminution of membrane lipid rafts and signaling lipids, and impairment of downstream PI3K activity, including epithelial growth factor (EGF)-receptor/ErbB/HER-function and expression. Marja Jäättelä underlined how lipid biosynthesis is needed for lysosomal stability, which is essential for autophagosome maturation and development of multidrug resistance in transformed cells. She found that lysosomal heat shock protein 70 (Hsp70) enhances the activity of lysosomal acid sphingomyelinase (ASMase). She is developing/testing ASMase- inhibiting cationic amphiphilic drugs, including antihistamines, as novel anticancer therapeutics with promising results, especially in patients with records of concurrent chemotherapy.

## pH Dynamics in Cancer

Jacques Pouysségur discussed therapeutic possibilities of a metabolic catastrophe when targeting both glycolytic and oxidative pathways by, e.g., combining inhibitors of monocarboxylate transporters (MCTs; preventing lactate efflux and stalling glycolysis) with metformin (targeting oxidative metabolism). He introduced transporters for essential nutrients as therapeutic targets, highlighting oncogene-regulated amino acid transporter SLC7A5. Silvia Pastoreková showed that carbonic anhydrase (CA)IX regulates metabolic adaptions in the hypoxic, acidic and nutrient-limited tumor microenvironment. CAIX engages in a feedback loop with hypoxia-inducible factor-1 (HIF-1) and regulates multiple metabolic enzymes, enabling cancer cell metabolic adaptability. Holger M. Becker demonstrated that lactate transport in cancer cells is decreased by CAII, CAIX or CAXII knockdown, but not by broad CA inhibition. CAs could thus act as “proton antennae” for MCTs, facilitating lactate-H^+^ transport non-catalytically and promoting cancer cell survival. Proffered paper/poster presentations offered novel insights into the frequent silencing of urea cycle enzyme ASS1 in cancer (Alon Silberman); Na^+^/H^+^ exchanger NHE1 in cancer cell motility (Laurent Counillon, Stephan J. Reshkin), dual regulatory roles of LDHB on prolylhydroxylase-2 and, thus, HIF-1 (Piotr Bański); metabolic effects of targeting MCT1 *in vivo* and *in vitro* (Valéry L. Payen); and MRI-CEST pH imaging in efficacy assessment of dichloroacetate treatment (Annasofia Anemone).

## Microenvironment and Stroma

This session was dedicated to the interplay between metabolism and the tumor microenvironment. Guy Eelen showed that (lymphatic) endothelial cells (ECs) use fatty acid-derived carbons for the production of deoxyribonucleotides for DNA synthesis. Massimiliano Mazzone then demonstrated that hypoxic tumor-associated macrophages (TAMs) upregulate REDD1, a negative mTOR regulator, which in turn hinders glycolysis and supports TAMs in generating abnormal blood vessels. In these communications, targeting EC and TAM metabolism was shown to normalize blood vessels and to reduce metastatic dissemination. Two other communications were dedicated to tumor acidosis. Cyril Corbet showed how acidosis induces a fatty acid-dependent metabolic shift in tumors associated with a global change in mitochondrial protein acetylation that, e.g., leads to partial electron transport chain complex I inhibition, thus preventing the production of reactive oxygen species (ROS). Sofia Avnet showed how acidic pH leads to the secretion of a cocktail of inflammatory and nociceptive mediators by mesenchymal cells of the bone marrow, which thereby contribute to cancer-associated bone pain. A proffered paper presentation by Silvia Lemma from the same group documented that lactate generated by cancer cells directly fuels the mitochondrial metabolism of osteoclasts and participates to bone resorption.

## Autophagy

Patrizia Agostinis reviewed recent findings indicating that increased autophagy negatively affects immunogenic cell death, that chloroquine reduces tumor hypoxia and improves chemotherapeutic efficacy through autophagy-independent vascular normalization, and that increased expression of BNIP3, a HIF-induced gene promoting autophagy, correlates with poor prognosis in melanoma. Nathalie M. Mazure then reported that BNIP3 and truncation of mitochondrial voltage-dependent anion channel 1 (VDAC1) contribute to apoptosis resistance in hypoxic cancers, which can be decreased by silencing p53. Carine Michiels showed that cytoprotective autophagy contributes to cancer resistance to taxol. Taxol indeed activates activating transcription factor 4 (ATF4), which is involved in taxol-induced autophagy and contributes to adaptation and resistance of breast cancer cells to chemotherapy in hypoxic tumors. Angelo De Milito showed how tumor acidosis mediates insensitivity of cancer cells to chloroquine. Salinomycin was identified as a potent cytotoxic agent preferentially killing cancer cells in acidic microenvironmental conditions due to increased intracellular accumulation of this strong autophagy inhibitor. Breast cancer stem cells (BCSC) are more sensitive to salinomycin than non-BCSC, and acidic conditions enhance the ability of salinomycin to inhibit mammosphere formation. Laura Brohée discussed the pro-tumorigenic roles of lipins and how lipin inhibitor propranolol increases the sensitivity of prostate cancer cells to 2-deoxyglucose by inhibiting autophagy.

## Epigenetics and Other Aspects of Tumor Metabolism

In this session, François Fuks summarized the current knowledge about DNA (hydroxyl)methylation and RNA modifications in cancer. This lecture was echoed by Manel Esteller who reviewed the epigenetic machinery (DNA methyltransferases, methyl-CpG-binding domain proteins, histone deacetylases, histone methyltransferases, histone demethylases and polycomb proteins) involved in the control of DNA methylation. Perturbations of these systems in cancer lead to abnormal methylation patterns not only in classical tumor suppressor genes but also in genes related to non-coding RNAs that possess growth inhibitory functions. Eyal Gottlieb then provided a causal link between mutations of metabolic enzymes and altered epigenetics by showing that loss of function mutations of succinate dehydrogenase (SDH) increase susceptibility to cancer by inhibiting α-ketoglutarate-dependent histone and DNA demethylases. Stine F. Pedersen focused on altered miRNA expression in breast cancer. She showed that upregulation of constitutively active HER2 receptor variant p95HER2 triggers miR-221/222 and miR-503 expression, which in turn inhibit the activity of MYB transcription factors and increase Na^+^-HCO_3_^-^-cotransporter mRNA stability. Cristovão M. Sousa concluded the session with new data indicating that stroma-associated pancreatic stellate cells fuel pancreatic cancer cells with alanine, thus revealing alanine as a TCA cycle fuel alternative to glucose and glutamine.

## Metabolic Control of Stemness and Metastasis

Aspects of the crosstalk between metabolism and cellular differentiation/metastasis were discussed. Based on previous work (Wanet et al., 2014), Patricia Renard elegantly showed the role of mitochondrial metabolism in mediating hepatocyte differentiation from bone marrow-derived mesenchymal stem cells. This team identified the interplay between hepatic differentiation and mitochondrial biogenesis, highlighting the role of transcription factors peroxisome proliferator-activated receptor gamma coactivator 1-α (PGC1-α) and HIF-1. Although these findings involved untransformed cells, their potential was also clear for cancer cells, with stemness as an important issue. Paolo E. Porporato then reported that mitochondrial metabolism controls cancer cell migration, invasiveness and metastasis through the production of mitochondrial ROS acting as signaling molecules (Porporato and Sonveaux, 2015). The perspective of combining specific chemotherapy regimens that induce moderate levels of mitochondrial ROS with targeted antioxidant therapies was discussed. Mojca Pavlin provided evidence that extreme impairment of metabolic fluxes by metformin treatment and glucose depletion triggers MDA-MB-231 breast cancer cell migration and increases their resistance to anoikis. Antoinette van Weverwijk identified by *in vivo* screening metabolic enzymes involved in breast cancer invasiveness. Myriam Y. Hsu showed that lactate transporter MCT1 promotes metastasis by a mechanism independent of its transporter activity.

## Therapy

Despite high incidence of chemoresistance, cisplatin still stands as the first-line agent for epithelial malignancies. Cisplatin cytotoxicity is due to the formation of DNA-adducts, but other mechanisms are involved, including interaction with phospholipids, proteins, RNA and mitochondrial DNA (mtDNA), leading to energetic dysfunctions. Monica Montopoli confirmed this hypothesis through metabolomic profiling of cisplatin-resistant cells that showed increased use of glucose and PPP metabolism. Chemosensitization may be achieved by limiting the enzymatic activity of glucose-6-phosphate dehydrogenase and the PPP. Anna Maria Porcelli underlined that energetic dysfunctions elicited by cisplatin can cause resistance following polychemotherapy with cisplatin and antimitogenic drugs like paclitaxel. Platinum adduct-mediated metabolic alterations indeed drive cells into a low-proliferative state. As for chemotherapy, cancer metabolism is an important determinant of cancer radiosensitivity, in that ionizing radiation is cytotoxic via ROS-dependent DNA damage and that mitochondria play a central role in cellular responses to redox stress. Debora Grasso showed that radioresistant head-and-neck carcinoma cells shift to a more oxidative metabolism compared to sensitive cells. Sven de Mey further showed that treatment of preclinical models of colorectal cancer with phenformin significantly radiosensitized cancer cells under hypoxia, possibly by improving tumor oxygenation via inhibition of mitochondrial complex I.

## Imaging

Imaging technologies provide a better understanding of the interplay between tumor behavior and its microenvironment and are becoming increasingly relevant for clinical settings. Studying cancer cell dissemination in metastasis using time-resolved live-cell and intravital microscopy, Peter Friedl showed that multicellular clusters migrate collectively, which leads to collective organ colonization. However, in conditions of severe hypoxia, these clusters dissociate, resulting in single cell migration. To be able to distinguish between directed and random cell migration, Tina Freisinger reported on applied time-lapse microscopy in Ibidi culture chambers in which the migratory behavior of cancer cells exposed to chemotactic gradients can be analyzed. Time-lapse microscopy is also the basis of IncuCyte Zoom, introduced by Toon De Roeve, with which images of cell cultures are collected in real-time over prolonged times and continuously evaluated for numerous cell processes, including proliferation, migration and stem cell differentiation. The fate of isotope-labeled molecules in tumors can be followed by positron emission tomography (PET). In a preclinical report by Vincent F. Van Hée, [^18^F]-3-fluorolactate served as a new tracer of lactate uptake by oxidative cancer cells and for monitoring uptake inhibition by anticancer drugs targeting the lactate transporter MCT1 *in vivo*.

## Biomarkers

Biomarkers, whether genomic, proteomic or imaging, are critical components for assessing metabolic compartmentation between cancer cells, stromal cells and crosstalk between cancer and stroma. Arkaitz Carracedo used cutting edge bioinformatics to uncover metabolic switches that cooperate with phosphatase and tensin homolog (PTEN) loss to promote prostate cancer aggressiveness and metastasis. Aziz Aiderus analyzed gene expression from a set of 973 ER+ breast cancers. He showed that increased expression of a fatty acid metabolic signature (including carnitine palmitoyltransferase CPT1) was associated with good prognosis, which was validated in an independent test set as well as in lung and gastric cancers. Mitochondrial biomarkers were discussed next. Giuseppe Gasparre introduced several lines of evidence that mtDNA mutations can be heterogeneous and, in each case, the site and amount of mutational burden could be correlated with a metabolic phenotype, raising the idea that mtDNA can be interrogated as a biomarker. Gyorgy Szabadkai developed a massively parallel biclustering analytical approach to investigate ∼1,000 nuclear encoded mitochondrial gene expression patterns (nMGEP) in relationship to metabolic phenotypes and found that nMGEPs were highly predictive of metabolic subtypes in Luminal A and B breast cancers.

## Conclusion

Metabolic plasticity is a major determinant of cancer progression supported by the expression in cancer cells of different enzyme and transporter isoforms that activate alternative metabolic pathways under hostile conditions, and by metabolic relationships established between cancer and stromal cells. One of the critical contributions of the tumor stroma was illustrated by Pawel Swietach in the closing lecture of ISCaM2016, which reported that, when cancer cells suffer for inadequate blood perfusion, stromal cells can handle metabolic wastes through a syncytial network of cytoplasms interconnected by gap junctions.

The international Society of Cancer Metabolism aims to promote interactions between basic and clinical researchers and between young and senior scientists, which was facilitated by the relatively small size of the meeting (189 participants) and its international composition (attendees represented 30 countries). Social activities were organized. Oral and poster communications were dispatched to sessions focusing on emerging aspects of cancer metabolism that were co-chaired by a principal and by a young investigator. Prizes were awarded to young investigators who delivered best scientific presentations. Oral presentation prizes went to Cyril Corbet (1st ISCaM prize), Alon Silberman (2nd Organizers’ prize) and Debora Grasso (3rd Organizer’s prize). Their studies are highlighted above. Poster presentations prizes went to Irena Roci (1st ISCaM prize for a study using isotope tracing for metabolic characterization of complex cancer cell populations), Myriam Y. Hsu (2nd EACR prize, study detailed above) and Maša Ždralević (3rd EACR prize for a study showing that co-silencing LDHA and LDHB enhances metformin cytotoxicity). In line with ISCaM’s objective to improve the outcome of cancer patients through the discovery and development of new anticancer drugs, several potential anticancer targets related to tumor metabolism were presented.

Conclusively, ISCaM2016 provided the audience with most recent discoveries on metabolic networks in cancer. This major topic of investigation will be discussed and updated at the ISCaM2017 meeting in Bertinoro, Italy, 19–21 October 2017^2^.

## Note

Authors belong to the Board of the International Society of Cancer Metabolism (ISCaM) and/or were local organizers of the ISCaM2016 meeting. All authors equally contributed to this manuscript.

## Author Contributions

All authors listed have made a substantial, direct and intellectual contribution to the work, and approved it for publication.

## Conflict of Interest Statement

The authors declare that the research was conducted in the absence of any commercial or financial relationships that could be construed as a potential conflict of interest. The reviewer MR and handling Editor declared their shared affiliation, and the handling Editor states that the process met the standards of a fair and objective review.

## Footnotes

1 http://www.iscams.org/

2 http://iscams.org/meeting/iscam2017-4th-annual-meeting-cancer-metabolism
